# Molecular identification of *Trypanosoma theileri* (Laveran, 1902) in cattle from two slaughterhouses in Ecuador and its relation with other haemotropic agents

**DOI:** 10.3389/fvets.2023.1153069

**Published:** 2023-06-23

**Authors:** María Augusta Chávez-Larrea, Cristina Cholota-Iza, Jorge Cueva-Villavicencio, Michelle Yugcha-Díaz, Jorge Washington Ron-Román, Andrea Rodríguez-Cabezas, Claude Saegerman, Armando Reyna-Bello

**Affiliations:** ^1^Research Unit of Epidemiology and Risk Analysis Applied to Veterinary Sciences (UREAR-ULiège), Department of Infections and Parasitic Diseases, Fundamental and Applied Research for Animal and Health (FARAH) Center, Faculty of Veterinary Medicine, University of Liège, Liège, Belgium; ^2^Grupo de Investigación en Sanidad Animal y Humana (GISAH), Carrera de Ingeniería en Biotecnología, Departamento de Ciencias de la Vida y la Agricultura, Universidad de las Fuerzas Armadas ESPE, Sangolquí, Ecuador; ^3^Grupo de Investigación en Sanidad Animal y Humana (GISAH), Carrera de Ingeniería Agropecuaria, Departamento de Ciencias de la Vida y la Agricultura, Universidad de las Fuerzas Armadas ESPE, Sangolquí, Ecuador

**Keywords:** bovine trypanosomosis, *Trypanosoma theileri*, lineage (ThI-Th-II), cattle, Ecuador, *Anaplasma marginale*, *Babesia*, *Trypanosoma vivax*

## Abstract

*Trypanosoma theileri* is a worldwide distributed haemoparasite that has been reported throughout the American continent in various species, including bovines, buffaloes and bats. In bovines, high incidence of *T. theileri* can be harmful when associated with other infections or under stress situations. There is little information on this hemoflagellate in Ecuador, which prompted the study and molecular identification of the trypanosomes collected in two slaughtering centers. Between February and April 2021, a total of 218 samples of bovine blood were collected in abattoirs located in the Andean region of Quito (*n* = 83) and in the coastal region, in Santo Domingo (*n* = 135). Quito public Slaughterhouse is the biggest in Ecuador, and for that, they receive animals from all country; on the other hand, Santo Domingo's Slaughterhouse is a small one where mainly females from the region are sacrificed and some males. The samples were evaluated using two molecular tests, the PCR cathepsin L-like (CatL) specific for *T. theileri* and for the positive samples, a Nested PCR that targets the ITS of the 18S gene. The corresponding PCR products were sequenced, analyzed by BLAST/NCBI and the sequences were used to build a concatenated phylogenetic tree, using the MEGA XI software. Overall, 34 out of the 218 samples, (15.6%) were positive to *T. theileri* by PCR CatL, resulting from 20/83 (24.1%) positives from the Quito abattoir and 14/135 (10.4%) from the Santo Domingo slaughterhouse. These prevalence rates were found to be significantly different (*p* = 0.006). According to the phylogenetic tree based on the CatL and ITS concatenated sequences (*n* = 13), the two novel Equatorial *T. theileri* isolates, ThI (*n* = 7) and ThII (*n* = 6) are closely related and associated to the IC, IB and IIB genotypes, present in Brazil, Venezuela and Colombia. Thirty-one out of the thirty-four *T. theileri-positive* bovines were co-infected with other haemotropic pathogens, *Anaplasma marginale Babesia* spp and *T. vivax*. This coinfection could be responsible for additional pathologies and harmful effects on the affected cattle. This study presents the molecular identification and genotypification of *T. theileri* isolated from cattle in Ecuador through the analysis of *CAtL* and *ITS* sequences, and the high frequency of coinfection of this hemoflagellate with other blood haemotropic organisms.

## 1. Introduction

In Ecuador, livestock production represents an important component of its economy with a 7.7% contribution to the Gross Domestic Product (GDP), and an estimated bovine population of 4.34 million that is distributed in four regions: 41.24% in the Coast region, 46.11% in the Mountain range (Andes), 9.65% in the Amazon and 0.43% in the Insular or Galapagos region (1). The division of Ecuador in four natural regions, coastal, Andes, Amazon (2) and Insular (Galapagos Islands) influences the distribution and management of various bovine breeds, as well as disease prevalence and disease risk factors (3).

Bovine trypanosomosis is a hemoparasitic disease distributed throughout parts of the African and American continents. In Latin-America, *Trypanosoma vivax, Trypanosoma evansi* and *Trypanosoma theileri* are the main trypanosome species that affect bovines (4). *T. theileri* is the least studied since it has been considered a nonpathogenic parasite (5). However, some recent studies show that *T. theileri* is an opportunistic parasite that can cause anemia, fever, swollen lymph nodes and lower hemoglobin concentration (6, 7).

*Trypanosoma theileri* has been classified within the Megatrypanum subgenera. It is larger than *T. evansi* and *T. vivax*, reaching a length between 69 to 109 μm it. This parasite has a free flagellum, with a well-developed undulating membrane and its posterior part is conical (4). The sequencing and subsequent transcriptome of *T. theileri* has revealed greater proximity to *Trypanosoma cruzi* and *Trypanosoma rangeli*, in the Stercoraria clade, as compared to *T. brucei* and *T. vivax*, which belong to the Salivarian clade. Like other trypanosomes, *T. theileri* persists in the host for a long time due to an efficient evasion mechanism that involves the synthesis of five diverse families of GPI-anchored surface proteins with conserved N- and C-terminal and over 1,000 genes that encode surface proteins distinct from the Variable Surface Glycoprotein (VSG) characteristic of the Salivarian group (8).

*T. theileri* has a cosmopolitan distribution throughout the world from Asia to America (5), infecting different species of the Order Artiodactyl, especially cattle and buffaloes (9). In Colombia, a northern neighbor country to Ecuador, the reported prevalence values for *T. theileri* appear to be higher in cattle (38.6 and 50.9%) than in buffaloes (28.2%) (9, 10). Lower prevalence values have been reported by Pacheco et al. (11), using a PCR based on Cathepsin L-like gene (PCR-TthCATL) in Brazil, with prevalence values of 42.19% per farm and 12.19% per animal.

The transmission of *T. theileri* can occur cyclically, mainly by horseflies, where the infective form is found in the intestine. This hemoflagellate like others within the Stercoraria group, is transmitted to the vertebrate host through contamination of wounds caused by horsefly bites (4, 10). Studies in Poland and Germany showed that 33.68 and 39% of *T. theileri* infected horseflies, respectively (12, 13). In Brazil, the reported prevalence of *T. theileri* in horseflies was 40 and 70% in two different geographical areas. In Ecuador there are few studies concerning tabanid fauna, however, in 2009 Cárdenas et al. (14) revealed a high density and species diversity for the country. In addition, iatrogenic and mechanical transmission by *Phlebotomus* (10, 15) and Aedes mosquitoes has also been reported (16).

The presence of trypanosomosis represents a limiting factor for livestock productivity due to economic losses attributed mainly to low milk production (17).

Despite this, there is little information on bovine trypanosomosis in Ecuador; however, Coello Peralta et al. found 20% positive samples of *Trypanosoma* spp. in sheep blood smears (18). Other studies by Medina-Naranjo et al. found a seroprevalence of 31.3% for *Trypanosoma* spp. using ELISA in the province of Pastaza (Amazon region) (19). In 2020 the first report of *T. vivax* was made in an outbreak in cattle in the Canton El Carmen, in the Manabí province, in the coastal region (20), and, regarding *T. theileri*, this hemoflagellate was recently described in the Ecuadorian Amazon region, with a prevalence of 11.4% (21).

Other homeotropic agents have also been reported in Ecuador; *A. marginale* was first described in the Santo Domingo Province after analysis by PCR of 151 blood samples, which resulted in a high, 86.1% rate of rickettsia infection (22). In Zamora-Chinchipe (South-Eastern Ecuador, close to Peru), a 68.8% prevalence was reported, indicating the widespread distribution and genetic variability of this bacterial pathogen in the country (21%) (23).

Even in the Galapagos Islands, this Rickettsia has been described, with a prevalence higher than 90%, which indicates the endemic nature of anaplasmosis in the islands (24). Regarding bovine babesiosis, Chávez-Larrea et al. (25) determined the presence of *Babesia* spp. by PCR in 18.94% (14.77% *Babesia bovis* and 4.17% *Babesia bigemina*) of twenty farms around El Carmen at 300 m.a.s.l.. Curiously, in this study, they found a prevalence of 20.28% (14.69% *B. bovis* and 5.59% *B. bigemina*) in Quito at 2469 m. a. s. l., demonstrating the adaptation of the vectors at higher altitudes (25).

The identification of the *Trypanosoma* species and its relationship with other haemotropic that are present in Ecuador, their distribution and prevalence are important aspects to clarify their epidemiology and to set the basis to implement timely and adequate diagnostic protocols and treatments. Similar studies led to the description of a novel trypanosome species, *Trypanosoma* (*Megatrypanum*) *trinaperronei* n. sp. in the white-tailed deer in Venezuela (26). For this reason, the present study focused, on the molecular identification of *T. theileri* in cattle originating in two slaughterhouses from the Pichincha and Santo Domingo de los Tsachilas provinces and its relationship with other haemotropic.

## 2. Materials and methods

### 2.1. Context of the study

**Figure 1:**
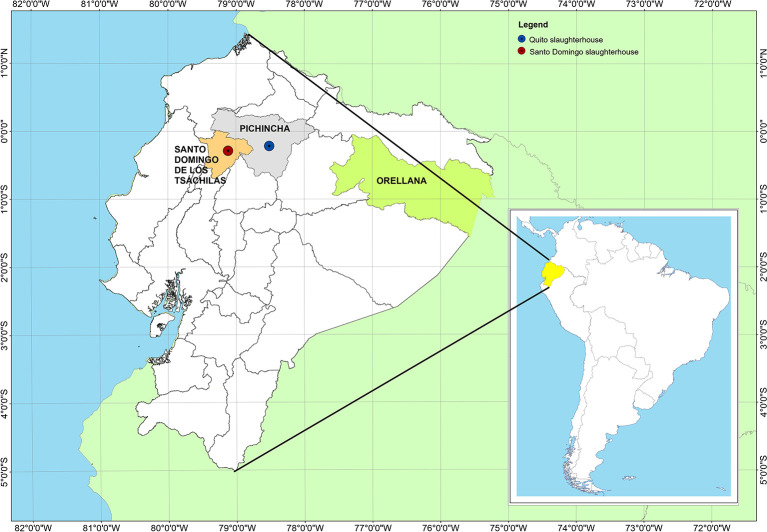
Location of the slaughterhouses of Quito and Santo Domingo and Provincia de Orellana in Ecuador.

To study the presence of *Trypanosoma theileri* in Ecuador, blood specimens were collected in the Quito and Santo Domingo slaughterhouses (Figure 1). Three interventions were done in the Quito slaughterhouses between February and March 2020, while four interventions were performed in the Santo Domingo abattoir between March and April 2021.

The Quito slaughterhouse is located in the capital city of Quito, in the Pichincha province, belonging to the Sierra (Andes) region, at 2.850 m above sea level. The Santo Domingo slaughterhouse is in the Santo Domingo de los Tsachilas province, 133 km from the city of Quito, at 655 m above sea level, in the Coastal zone (Figure 1).

### 2.2. Sampling and conservation of bovine blood samples

A total of 218 cattle were sampled, including two species: *Bos indicus* and *Bos taurus*. In the slaughterhouses of Quito, *n* = 83, and Santo Domingo, *n* = 135. Blood samples were collected in 10 mL tubes with EDTA from the jugular vein at the time of the sacrifice of the animals. Depending on the number of animals slaughtered in the Quito slaughterhouse (27), one out of 10 animals was selected for each intervention. Given the low number of animals slaughtered daily, one out of every two animals was sampled for each intervention in the Santo Domingo slaughterhouse.

The Quito slaughterhouse is one of the largest in Ecuador, where many male and female animals are slaughtered. On the other hand, the Santo Domingo slaughterhouse is small, where mainly females from surrounding areas are killed. The males in the area are transferred to the slaughterhouse in Quito or another private.

Samples were temporarily stored at room temperature while transferred to the laboratory and stored at−20°C at the Animal Biotechnology Laboratory of the Universidad de las Fuerzas Armadas ESPE and assigned identification numbers with information on sex and breed.

### 2.3. Packed cell volume and hematocrit concentration technique

The PCV value of the blood samples was determined using capillary tubes with heparin (TECNAN, Navarra, Spain). The tubes were centrifuged (TG12M Madell Technology Corporation, Riverside, California, USA) at 12.000 rpm for 5 min. To evidence the parasite in the blood, within the first 4 h of sample collection, the heparinized capillary tubes were observed under a microscope (KRUSS, Hamburg, Germany) according to the HCT technique (28).

### 2.4. DNA extraction

Deoxyribonucleic Acid (DNA) extraction from blood samples was performed using the Genejet Whole Blood Genomic DNA Purification Mini Kit (Thermo Scientific, Waltham, Massachusetts, U.S.). The DNA integrity was verified on a 0.8% agarose gel and DNA concentration was quantified by UV spectrophotometry, using the NanoDrop 2000 (Thermo Fisher Scientific, Waltham, Massachusetts, USA).

### 2.5. Molecular diagnosis

Several primer sets were used to diagnose haemotropic pathogens (Table 1) and all PCR reactions were carried out in 25 μl reaction volumes, using the Proflex Thermal Cycler (Life Technologies, Carlsbad, California, U.S.A). The positive controls for *T. theileri* were the samples that tested positive for HCT. On the other hand, for Babesia, *T. vivax*, and *A. margianle*, previously prepared plasmids were used (29–31).

**Table 1:** List of primers used for molecular analysis by PCR for the detection of haemoparasites in cattle.

**Haemoparasites**	**Target gene**	**Primer**	**Oligonucleotide sequence (5^′^-3^′^)**	**Reference**
*T. theileri*	*CATL*	TthCATL1	CGTCTCTGGCTCCGGTCAAAC	(29)
		DTO155	TTAAAGCTTCCACGAGTTCTTGATGATCCAGTA	(30)
	*ITS*	ITS1	GAT TAC GTC CCT GCC ATT TG	(31)
		ITS2	TTG TTC GCT ATC GGT CTT CC	
		ITS3	GGA AGC AAA AGT CGT AAC AAG G	
		ITS4	TGT TTT CTT TTC CTC CGC TG	
*T. vivax*	*CATL*	TviCatL1	GCCATCGCCAAGTACCTCGCCGA	(30)
		DTO155	TTAAAGCTTCCACGAGTTCTTGATGATCCAGTA	
*T. evansi*	*ESAG*	ESAG6	ACATTCCAGCAGGAGTTGGAG	(32)
		ESAG7	CACGTGAATCCTCAATTTTGT	
*A. marginale*	*msp5*	19A	GTGTTCCTGGGGTACTCCTA	(22, 33)
		19B	TGATCTGGTCAGCCCCAGCT	
*B. bovis*	*RAP-1*	BoF	CACGAGGAAGGAACTACCGATGTTGA	(34)
		BoR	CCAAGGAGCTTCAACGTACGAGGTCA	
*B. bigemina*	*HYP*	BiIA	CATCTAATTTCTCTCCATACCCCTCC	(35)
		BiIB	CCTCGGCTTCAACTCTGATGCCAAG	

### 2.6. Detection of *T. theileri*

A specific catalytic domain of cathepsin L-like (*CATL*-like) PCR, that amplified a partial sequence of the *CATL* gene (29), was performed to detect the presence of *T. theileri*, using between 100 and 150 ng of DNA, as modified by Yokoyama et al. (36). Samples that were positive for the *CATL*-like PCR were analyzed with a Nested PCR based on the internal transcribed spacers (*ITS*) region, as described by Cox et al. (31), except for the temperature of annealing which was increased 59°C to improve specificity. This genetic marker was used to distinguish the *T. theileri* lineages and phenotypes. The first-round reaction mixture for the nested PCR contained 100–150 ng of DNA and the external primers ITS1 and ITS2, while the second round used 1 μL of the PCR product from the first reaction and the ITS3 and ITS4 internal primers (Table 1).

### 2.7. Detection of coinfection with other haemotropic agents

All positive samples for the *CATL-*like PCR were further analyzed with different PCR assays to determine coinfection. For *T. vivax*, a specific *CATL*-like PCR was performed (20, 30), for *T. evansi*, the ESAG primer set was used (32), for *A. marginale* the *msp5* PCR was utilized (22, 33), while *B. bovis* and *B. bigemina* were diagnosed by *RAP-1* PCR and *HYP* PCR, respectively (34, 35).

### 2.8. Phylogenetic analysis

Positive PCR products for *CATL*-like PCR specific for *T. theileri* and positive PCR products for ITSs Nested PCR, were sequenced in (Macrogen, Seoul, Korea), using the Sanger technique. The consensus sequences were deposited in GenBank (Accession numbers for *CATL*-like: OQ304106, OQ304107, OQ304108, OQ304109, OQ304110, OQ304111, OQ304112, OQ304113, OQ304114, OQ304115, OQ304116, OQ304117, OQ304118 and Accession numbers for ITS: OQ341204, OQ341205, OQ341206, OQ341207, OQ341208, OQ341209, OQ341210, OQ341211, OQ341212, OQ341213, OQ341214, OQ341215, OQ3412166). The similarity of the consensus sequences obtained for both the *CATL*-like and the *ITS* were analyzed with the BLAST tool (Basic Local Alignment Search Tool the National Center for Biotechnology Information). Phylogenetic relationships were based on the sequences obtained in this study and on previously described sequences available in the GenBank database (9, 30, 37). The construction of a Maximum Likelihood tree, Tamura-Nei model (38) was carried out using the MEGAXI program.

### 2.9. Epidemiological and statistical analysis

A Microsoft Excel sheet was used to organize, clean, and validate the laboratory results. Significant differences in the distribution of *T. theileri* lineages and biotypes between the two abattoirs, between individuals of different sex and animal breeds, were analyzed by the Fisher's exact test, a statistical test used to determine nonrandom associations between a small group of observations. Bovine origin was excluded in the analysis of the Santo Domingo abattoir, since that information was not considered reliable.

## 3. Results

Out of the 218 analyzed samples, 38.1% were collected in the Quito slaughterhouse, while the remaining 62% were from the Santo Domingo slaughterhouse. Regarding the origin of the animals, we relied only on the information collected in the Quito slaughterhouse, where 11/83 (13.3%) of the animals originated in the Santo Domingo Province, 13/83 (15.7%) in the Orellana province, and 59/83 (71.1%) with unknown provenance. The results of the diagnostic tests regarding the sex variable, were only analyzed for the Quito slaughterhouse, where the sampling included similar percentages of males (44.6%) and females (55.4%). By contrast, an unintentional bias was introduced in the sampling of slaughter groups at the Santo Domingo abattoir, with a very low percentage of males (3%), as compared to females (97%).

From the 218 total bovine samples, 13 (6.0%) were positive with *Trypanosoma* spp. by the HCT technique, corresponding to a significant difference (*p* < 0.05) in the prevalence (14.5%) in the Quito abattoir, as compared to the prevalence (0.74%) in the Santo Domingo slaughterhouse. The average values of the packed cell volume (PCV) for the tested animals and those positive to *T. theileri* were 37.7 and 38.42%, with standard deviations of 10.0 and 7.6%, respectively.

The *CATL*-like PCR test identified 34 samples positive to *T. theileri* (15.6%), 20 (24.1%) originating in the Quito abattoir and 14 (10.4%) in the Santo Domingo slaughterhouse, with a significant difference between these locations (*p* = *0.006*). Among the 20 samples that were positive by CATL-like PCR in the Quito abattoir, there were 10 males (27.0%) and 10 females (21.7%), which resulted in no significant difference in the sex variable (*p* > 0.05). In the Quito abattoir, no statistically significant differences were found among the bovines positive to *T. theileri* and the two species that were examined, *Bos indicus* and *Bos taurus*.

The use of the *CATL*-like PCR showed that 7 of the 13 HCT-positive bovines, corresponded to *T. theileri* infections and 5 to *T. vivax* infections. Through the use of molecular tests, the prevalence of five haemotropic agents was obtained: *T. theileri* (15.6%)*, T. vivax* (7.3%), *A. marginale* (60.1%), *B. bovis* (3.2%) *and B. bigemina* (2.3%). No animals were positive to *T. evansi*, using the ESAG PCR. Table 2 presents distribution details of the positive cases of the various haemopathogens that were analyzed, regarding the two abattoirs, sex and species of the sampled individuals.

**Table 2:** Distribution of positive cases for *T. theileri* and other haemopathogens in the slaughterhouses of Quito and Santo Domingo.

**Parameter**	**Total number of animals (%)**	**Positive HCT**		**Positive** ***T. theileri***		**Positive** ***T. vivax***		**Positive** ***A. marginale***		**Positive** ***B. bovis***		**Positive** ***B. bigemina***	
				**PCR** ***CATL*** **-like**		**PCR** ***CATL*** **-like**		**PCR** ***Msp5***		**PCR** ***RAP-1***		**PCR** ***HYP***	
		**No**	**95 % (IC)**	*N*°	**95 % (IC)**	*N*°	**95 % (IC)**	*N*°	**95 % (IC)**	*N*°	**95 % (IC)**	*N*°	**95 % (IC)**
Quito	**83 (38.1)**	**12**	**14.5 (6.9–22.0)**	**20**	**24.1 (15.4−34.7)**	**11**	**13.3 (6–20.6)**	**61**	**73.5 (64.0–83)**	**4**	**4.8 (0.2–9.4)**	**3**	**3.6 (0.8–10.2)**
**Sex**													
Male	37 (44.6)	5	13.5 (2.5–24.5)	10	27.0 (12.7–41.3)	5	13.5 (2.5–24.5)	24	64.9 (49.5–80.3)	0	0.0	0	0.0
Female	46 (55.4)	7	15.2 (4.8–25.6)	10	21.7 (9.8–33.7)	6	13.0 (3.3–22.9)	37	80.4 (69.0–91.9)	4	8.7 (0.6–16.8)	3	6.5 (1.4–17.9)
**Species**													
Bt + Bi	21 (25.3)	2	9.5 (1.2–30.4)	5	23.8 (5.6–42.03)	1	4.8 (0.1–23.8)	15	71.4 (52.1–90.8)	1	4.8 (0.1–23.8)	0	0.0
Bt	52 (62.7)	8	15.4 (5.6–25.2)	13	25.00 (13.2–36.7)	9	17.3 (7.0–27.6)	37	71.2 (58.8–83.5)	3	5.8 (1.2–16.0)	3	5.8 (1.2–16.0)
Bi	10 (12.1)	2	20.0 (2.5–55.6)	2	20.0 (2.5–55.6)	1	10 (0.3–44.5)	9	90 (71.4–100.0)	0	0.0	0	0.0
Santo Domingo	**135 (61.9)**	**1**	**0.7 (0.02–4.1)**	**14**	**10.4 (5.8–16.8)**	**5**	**3.7 (0.5–6.8)**	**70**	**51.9 (43.4–60.3)**	**3**	**2.2 (0.5–6.4)**	**2**	**1.5 (0.2–5.2)**
**Sex**													
Male	131 (97.1)	1	0.8 (0.02–4.2)	13	9.9 (4.8–15.0)	4	3.1 (0.1–6.0)	66	50.4 (41.9–58.9)	3	2.3 (0.5–6.5)	2	1.5 (0.2–5.4)
Female	4 (3.0)	0	0.0	1	25 (0.6–80.6)	1	25 (0.6–80.6)	4	100	0	0.0	0	0.0
**Species**													
Bt + Bi	68 (50.4)	0	0.0	6	8.8 (2.1–15.6)	2	2.9 (0.4–10.2)	34	50 (38.1–61.9)	3	4.4 (0.9–12.4)	2	2.9 (0.4–10.2)
Bt	52 (38.52)	0	0.0	6	11.5 (2.9–20.2)	2	3.9 (0.5–13.2)	30	57.7 (44.3–71.1)	0	0.0	0	0.0
Bi	15 (11.1)	1	6.7 (0.2–32.0)	2	13.3 (1.7–40.5)	1	6.7 (0.2–32.0)	6	40 (15.2–64.8)	0	0.0	0	0.0
TOTAL	**218**	**13**	**6** (3.2–10.0)	**34**	**15.6 (11.1–21.11)**	**16**	**7.3 (3.9–10.8)**	**131**	**60.1 (53.6–66.6)**	**7**	**3.2 (0.9–5.6)**	**5**	**2.3 (0.3–4.3)**

Bt, Bos Taurus; Bi, Bos indicus; IC, confidence interval. The bold values indicate the total numbers of each slaughterhouse and the totals of the sampling.

All the *T. theileri* positive bovines (*n* = 20) in the Quito slaughterhouse were coinfected, 15 (18.1%) with *A. marginale* and 5 (6.0%) with *T. vivax* and *A. marginale*. With regard to the Santo Domingo slaughterhouse 11 of the 14 bovine samples that were positive to *T. theileri* showed coinfection; 6 (4.4%) with *A. marginale*, 2 (1.5%) with *T. vivax* and 3 (2.2%) with *T. vivax* and *A. marginale* (Table 3).

**Table 3:** Details of *T. theileri* coinfections with other haemopathogens in the slaughterhouses of Quito and Santo Domingo.

**Slaughterhouse**	***T. theileri* (PCR *CATL* Like)**	***T. vivax* (PCR *CATL* Like)**	***A. marginale* PCR *msp5***	***B. bovis*** **PCR *RAP-1***	***B. bigemina* PCR *HYP***	**Total *N*°(%)**
**Quito**						
	**-**	**-**	**-**	**-**	**-**	21 (25.3)
	**-**	**-**	**+**	**-**	**-**	32 (38.6)
	**-**	**-**	**+**	**-**	**+**	1 (1.2)
	**-**	**-**	**+**	**+**	**-**	1 (1.2)
	**-**	**-**	**+**	**+**	**+**	2 (2.4)
	**-**	**+**	**-**	**-**	**+**	1 (1.2)
	**-**	**+**	**+**	**-**	**-**	5 (6.0)
	**+**	**-**	**+**	**-**	**-**	15 (18.1)
	**+**	**+**	**+**	**-**	**-**	5 (6.0)
**Santo Domingo**						
	**-**	**-**	**-**	**-**	**-**	59 (43.7)
	**-**	**-**	**-**	**+**	**+**	1 (0.7)
	**-**	**-**	**+**	**-**	**-**	59 (43.7)
	**-**	**-**	**+**	**-**	**+**	1 (0.7)
	**-**	**-**	**+**	**+**	**+**	1 (0.7)
	**+**	**-**	**-**	**-**	**-**	3 (2.2)
	**+**	**-**	**+**	**-**	**-**	6 (4.4)
	**+**	**+**	**-**	**-**	**-**	2 (1.5)
	**+**	**+**	**+**	**-**	**-**	3 (2.2)

+ Positive and − Negative.

**Figure 2:**
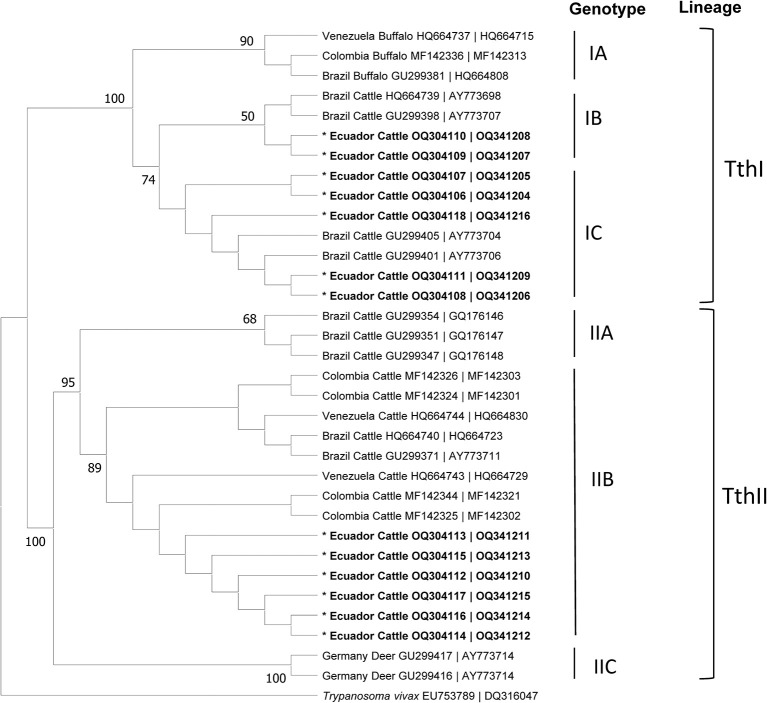
Maximum Parsimony Phylogram built with concatenated sequences of CatL gene and ITS region of *Trypanosoma theileri*, **Lineages**: TthI and TthII. **Genotypes**: IA, IIA, IB, IIB, IC, IIC. The DNAs obtained from cattle of two slaughterhouses in Ecuador in bold.

The phylogenetic tree constructed from the 13 best quality sequences (Figure 2) of the *CATL*-like and ITS PCR amplicons (*n* = 24), showed that the *T. theileri* isolates cluster within the ThI (*n* = 7) and ThII (*n* = 6) lineages. In the Quito slaughterhouse, two samples belong to the IB genotype and three to the IC genotype, within the TthI lineage, while five samples belonged to the IIB genotype in the TthII lineage. On the other hand, in the Santo Domingo slaughterhouse, two samples aligned with genotype IC of the TthI lineage, and one sample with the IIB genotype of the TthII lineage. The phylogeographic tree (Figure 2) showed that seven of the isolates, from Quito (*n* = 5) and Santo Domingo (*n* = 2), clustered within the Brazil clades; while the 6 remaining isolates, from Quito (*n* = 5) and Santo Domingo (*n* = 1), clustered with the Venezuelan and Colombia clades.

Out of the 13 samples that were analyzed for lineages and genotypes, 12 showed coinfection with *T. vivax* and/or *A. marginale*. Further details of the lineages-biotypes and coinfection with *T. vivax, A. marginale, B. bovis* and *B. bigemina* are presented in Table 3.

As for the origin of the animals from the Quito slaughterhouse, it was possible to determine that TthI-IB (*n* = 2) and TthI-IC (*n* = 2) originated from the province of Santo Domingo de los Tsachilas, while the animals with the genotype TthII-IIB (*n* = 5) originated from the province of Orellana.

## 4. Discussion

This study evidenced the presence of *T. theileri* in bovines from two slaughterhouses in Ecuador, using a molecular test (*CATL*-like PCR). Significant prevalence values of 24.1 and 10.4% for *T. theileri* were determined for the Quito and the Santo Domingo abattoirs, located in the Andean and Coastal regions, respectively.

Prevalence values for *T. theileri* in South America are high, suggesting that this parasite is widely distributed in the cattle production systems in this region. The global prevalence values (15.6%) determined in the present study is congruent with reports from some farms in neighboring countries, including 5.0 and 30.4% in Venezuela and Brazil, respectively (39), 8.1% in Brazil (11), and 38.6 to 50.3% in Colombia (6, 9). The high prevalence values for *T. theileri* in other countries have been related to environmental conditions, host, and parasite factors (9). This report of the presence of *T. theileri* in cattle in Ecuador highlights the importance of these neglected diseases and the need to focus more attention and resources on haemopathogen agents that affect the cattle industry in this country and region.

Prevalence values obtained from the two slaughterhouses, in Quito and Santo Domingo, evidenced the presence of several haemopathogens, including in decreasing order: *A. marginale* (60.1%, the most predominant), *T. theileri* (15.6%)*, T. vivax* (7.3%), *Babesia bovis* (3.2%), *B. bigemina* (2.3%) and *T. evansi* (0 %). Our study did not evidence *T. evansi* in any of the bovine samples from the two slaughterhouses. These values are compatible with previous studies in Ecuador, 31.0% for *Trypanosoma spp* and 65.5% for *A. marginale* in the Pastaza province (19) and 86.1% in the Santo Domingo de los Tsachilas province (22), 18.94 and 20.28% for *Babesia spp* in the Pichincha and Manabí provinces, respectively (25).

The implementation of molecular diagnostic tests such as the *CATL*-like PCR for *T. theileri* and *T. vivax* and *msp5* PCR for *A. marginale* showed mixed infections of *T. theileri* with *A. marginale*, as well as *T. theileri, T. vivax* and *A. marginale* in the Quito (*n* = 20) and Santo Domingo (*n* = 14) abattoirs. Coinfections with other haemotropics have been reported in Colombia, where 53.9% of the bovines present mixed infections and 26.7% presented coinfections of *T. theileri* with *A. marginale* (6). In another study, 83.3% of the bovines positive to *T. theileri* showed coinfection with other haemopathogens (36). All the animals infected with the genotyped *T. theileri* carried mixed infections, which reinforces the hypothesis that we are in the presence of an opportunistic parasite (9).

Although this study did not present a clinical assessment of the animals in the abattoirs, PCV analysis showed normal levels, above 24%, in all the bovine specimens, including those in the animals positive to *T. theileri*. This is in agreement with previous reports that show that bovines infected with *T. theileri* do not suffer from anemia and have normal levels of erythrocytes (40, 41). However, in spite of this, *T. theileri* can be considered a potentially pathogenic parasite, in association with other haemopathogens, as evidenced in a livestock area in Colombia, where *T. theileri* infection was higher in bovines with signs of anemia (9). Furthermore, *T. theileri* infections may result in chronic and mild clinical signs, with low and persistent parasitemias (8), as could be the case for the bovines that were included in this study, whose hematocrit levels were above 24%. These different pieces of evidence highlight the need to further investigate the possible link between of coinfection with *T. theileri* and other haemopathogens and clinical disease.

The prevalence of *T. theileri* based on the HCT (14.5 %) and PCR *CATL*-like (24.1%) tests was higher in the Quito abattoir as compared to the Santo Domingo slaughterhouse, with 0.7 and 10.4% prevalence values, respectively. The bovines positive to *T. theileri* (20/83), in the Quito abattoir originated in the Santo Domingo province in the Coastal region and in the Orellana province in the Amazon region. The difference in the distribution of the positive results of HCT suggests that bovine trypanosomosis in the Coastal region is endemic, unlike the Amazon region where disease outbreaks can occur. This proposal is reinforced by the recent finding of a large percentage of *Trypanosoma* spp. positive farms (8/32) in the Amazon region, using the HCT (Chávez- Larrea et al. data not published). This could be related with the great diversity of animal reservoirs, both livestock and/or wildlife in the Amazon region.

Statistical analysis of the *T. theileri* positive animals from the Quito slaughterhouse showed no significant differences concerning bovine species, nonetheless significant differences were observed in relationship to sex, with a higher prevalence in males. Even though creole cattle breeds could be a risk factor associated with the presence of *Trypanosoma* spp., as suggested by Jaimes-Dueñez et al. (42) in Colombia studying creole races, additional research is needed to clarify the role of other risk factors, including sex and host species.

*T. theileri* has been reported in various animal species including bovines, buffaloes (Asia, South America), antelopes (Africa) (4), horse in Malaysia (43) and bats (44), with several lineages and genotypes. The implementation of molecular diagnostic techniques using CatL and ITS fragments, followed by DNA sequencing and analysis have been previously used to analyze *T. theileri* genotypes (39, 40, 45). The two *T. theileri* lineages, Tth I and TthII identified in this study have been reported in other South American countries, including Colombia (9), Brazil and Venezuela (29, 39, 46). Several genotypes have been identified within these lineages as specific for each host species, such is the case for bovines (IIA, IB, IIB, IC), buffaloes (IA) and deers (IIC) (9, 29, 46). Genotypes IB, IIB and IC, which have been previously described in Brazil, Venezuela and Colombia (9, 11, 39) were identified in this study. Genotype IIB was the most prevalent in the samples that were analyzed in this study (6/13), which is also consistent with the most prevalent genotype in South America (9, 39). Likewise, in the study carried out by De la Cadena et al. (21), in the Ecuadorian Amazon, they point out the presence of the TthI and TthII genotypes using the cathepsin L-like and 18S ribosomal DNA for the phylogenetic tree.

The *T. theileri* sequences from the Quito and Santo Domingo slaughterhouses were placed within the clade of the Colombian isolates. It is possible that cattle displacement between the two countries might be responsible for the introduction of *T. theileri* to Ecuador. Very little is known about the origin of the first bovines that arrived in Ecuador. According to a study on the indigenous breeds, the first bovines being introduced in the XV century during the Spanish colonization, with some were introduced from the Pacific coast of Colombia and Panama and some from Rio de la Plata (47). This mobilization of cattle appears to have influenced the distribution of genotype IIB, which is the most disseminated in South America from cattle of Iberian origin (9). The other genotypes found in our study, IB and IC, like those found in Brazil, maybe were introduced more recently (9).

In Ecuador, the regions below 1,000 masl are found both in the coastal and Amazon regions. These two zones are separated by the Sierra region (Los Andes), where the heights range from 2,000 to 4,000 meters above sea level. For this reason, in this work, only animals positive for *T. theileri* were found in the coastal region (Province Santo Domingo de los Tsachilas) and the Amazon region (Province Orellana) (Figure 1).

In America, the transmission of *Trypanosoma* spp in bovine cattle is mainly mechanical, through Tabanids and other blood-sucking flies (4). Tabanids also appear to play an essential role in the transmission of *A. marginale* too (48). In the present study, the estimated prevalence for *A. marginale* was 38.6% in the Quito slaughterhouse and 43.70% in the Santo Domingo slaughterhouse. Most of the animals were coinfected with this bacteria, possibly due to high prevalence of *A. marginale* all over the country. There have not been any studies in Ecuador on the importance of Tabanids and other blood-sucking flies as vectors for these diseases in livestock. The presence of the *T. theileri* genotypes has also been shown in Tabanids, which reinforces their role in the dissemination of *Trypanosoma* spp in America (46).

## 5. Conclusions

This study constitutes the first report of lineages TthI and TthII and genotypes IB, IC and IIB of *T. theileri* in bovine cattle in Ecuador (Figure 2), a finding that could be important to establish the genetic relationship between the variants present in this country and others in the region. On the other hand, existing coinfections between *T. theileri, T. vivax, A. marginale, B. bovis* and *B. bigemina* were also evaluated at the slaughterhouse level.

This study underscores the presence of *T. theileri* in livestock in Ecuador, with a prevalence of 15.6% (34/218). Even though, *T. theileri* can be considered non-pathogenic or mildly pathogenic, further studies are needed to evaluate the epidemiological situation in the region and its true impact on the health of bovines and other susceptible species, especially in case of coinfection with other haemopathogens.

## Data availability statement

The original contributions presented in the study are included in the article/supplementary material, further inquiries can be directed to the corresponding author.

## Ethics statement

Ethical review and approval was not required for the animal study because the work was carried out with animals from slaughterhouses, after their sacrifice. Written informed consent was obtained from the owners for the participation of their animals in this study.

## Author contributions

Conceptualization: MC-L, CS, and AR-B. Methodology, data curation, and writing—original draft preparation: MC-L. Validation: MC-L, JC-V, AR-C, and CC-I. Formal analysis: MC-L, CC-I, and JR-R. Investigation: MC-L and AR-B. Writing—review and editing: AR-B, JR-R, and CS. Supervision: AR-C and CS. Project administration: JR-R and AR-B. Funding acquisition: CS, JR-R, and AR-B. All authors have read and agreed to the published version of the manuscript.
